# Non-infectious thrombotic endocarditis associated with chronic rheumatic heart disease and disseminated tuberculosis

**DOI:** 10.4322/acr.2021.269

**Published:** 2021-05-06

**Authors:** Aravind Sekar, Sanjeev Naganur

**Affiliations:** 1 Post Graduate Institute of Medical Education and Research, Department of Histopathology, Chandigarh, India; 2 Post Graduate Institute of Medical Education and Research, Department of Cardiology, Chandigarh, India

**Keywords:** Endocarditis, Non-Infective, Rheumatic Heart Disease, Tuberculosis

## Abstract

Rheumatic heart disease is still common in developing countries and requires prompt intervention to prevent chronic complications. Vegetations in rheumatic heart disease might be due to acute episodes of rheumatic fever itself or due to either infective endocarditis (IE) or Non-infectious thrombotic endocarditis (NITE). Each form of vegetations has specific pathological characteristics on gross and microscopic examination. However, clinically IE and NITE may have overlapping signs and symptoms. A chance of misdiagnosis of NITE as culture-negative infective endocarditis is higher if the former present with infective symptoms like fever. NITE of valves can be due to underlying associated malignant neoplasm, particularly mucinous adenocarcinoma, pneumonia, cirrhosis, autoimmune disorders, and hypercoagulable state. The coexistence of tuberculosis, non-infectious thrombotic endocarditis and rheumatic valvular heart disease was rarely documented in medical literature. We describe a case of chronic rheumatic heart disease with vegetations in the posterior mitral valve leaflet, treated as culture-negative infective endocarditis, which, at autopsy, reveals the presence of Nonbacterial thrombotic endocarditis vegetation over calcified, fibrosed mitral valve leaflets and associated disseminated tuberculosis along with classic pathological sequela findings of chronic rheumatic mitral valvular heart disease in lungs and liver.

## INTRODUCTION

Chronic rheumatic valvular heart disease is a worrisome complication of rheumatic fever, particularly in developing countries, and requires prompt surgical intervention to prevent further sequela of disease.^1^ Broadly, vegetative endocarditis is of four types such as rheumatic vegetations, infective endocarditis, non-infective thrombotic endocarditis (NITE), and Libman-sacks endocarditis. Each one has characteristic gross and microscopic findings. Rheumatic vegetations are pinhead-sized and usually found at the line of closure of both leaflets during acute rheumatic fever events. Infective endocarditis vegetations are bulky and seen over either normal or deformed valve leaflets. NITE vegetations are small bland vegetations and are often seen in debilitated patients due to sepsis or malignancy. Libman-sacks vegetations are seen both inflow and the non-flow surface of leaflets in cases of lupus erythematosus. We describe an autopsy case of chronic rheumatic heart disease associated with non-infectious thrombotic endocarditis over damaged posterior mitral valve leaflet and disseminated tuberculosis

## CASE STUDY

A 35-year-old man with a prior clinical diagnosis of rheumatic heart disease with severe mitral stenosis presented to the outpatient department with complaints of worsening dyspnea. He could not be submitted to mitral valve replacement due to financial constraints and was on medical management for 4 years. He also complained of 2-3 episodes of paroxysmal nocturnal dyspnea and orthopnea for 2- 3 days. He also had low-grade intermittent fever associated with general malaise for one month, which increased to a high grade for 2-3 days. He denied cough with expectoration. On general examination, he was thin built, anemic with obvious pedal edema; no cervical or axillary lymphadenopathy; jugular venous pressure was elevated; blood pressure was 90/60 mm Hg; pulse rate 96/min regular; respiratory rate- 16/minute; room air oximetry of 98%; Glasgow coma scale of 15. The pupils were Round, regular and reactive to light; Fundus was normal. On cardiovascular examination the first sound was loud, the second sound was narrowly split with a loud pulmonary component. There was no third heart sound. There was a sharp opening snap (OS) with a short A2-OS interval. At the apex, there was a grade 3/6 mid-diastolic murmur with a presystolic intensification, best heard with the bell of the stethoscope, in the left lateral position with the breath held in expiration. The respiratory system examination showed air entry bilaterally equal, bilateral basal crackles present. The abdomen examination depicted a hepatomegaly of 3-4 cm below right costal margin. The spleen was not palpable, and ascites was present. The neurological examination was normal. The laboratory findings at admission showed Hemoglobin 8.4 gm/dl (reference range [RR]; 13.8-17.2 gm/dL); total leukocyte count of 15700 cell/mm3 (RR; 4500-11000 cells/mm3); platelets of 346x103 cells/mm3 (RR;150-450x103 cells/mm3); serum creatinine-1.3 mg/dL (RR;0.8-1.2 mg/dL); serum albumin-2.8mg/dL (RR; 3.4-5.4 mg/dL); AST/ALT-35/24IU/L (RR; 7-55/8-48 U/L); CRP: 90.16 mg/L (Normal:<3mg/L); Procalcitonin: 0.647 ng/mL (RR; 0.1-0.49 ng/mL). Since infective endocarditis was suspected, blood culture was sampled thrice on subsequent days, which were all negative. Chest X-ray showed bilateral basal infiltrates (suggestive of severe pulmonary oedema). A 2D-ECHO showed severe mitral stenosis (MS), mitral valve gradient of 20/13 at 70 bpm, mitral valve area of 0.4sq cm, Mild mitral regurgitation/tricuspid regurgitation; Right ventricular systolic pressure-Right atrial pressure + 74 mmHg; no clot; normal left ventricular systolic function. Transesophageal echocardiography showed severe mitral stenosis with 7.6 x 11.4 mm vegetation on the posterior mitral leaflet, no intracardiac clot. Thus, a diagnosis of acute decompensated heart failure with culture-negative infective endocarditis was made, and hence started on parenteral antibiotics (Vancomycin and Ceftriaxone) with decongestive measures for heart failure. He was planned for mitral valve replacement. But he developed worsening of dyspnea with orthopnea along with new-onset fever associated with chills; He was initially managed on a line of cardiogenic shock with pulmonary edema; started on inotropic support along with non-invasive ventilation His shock was worsening and mean arterial pressure failed to normalize despite three inotropes at maximal dosage Despite all supportive measure his vitals continued to decline and he went into asystole. The resuscitation was unsuccessful and could not be revived.

## AUTOPSY FINDINGS

**Figure 1:**
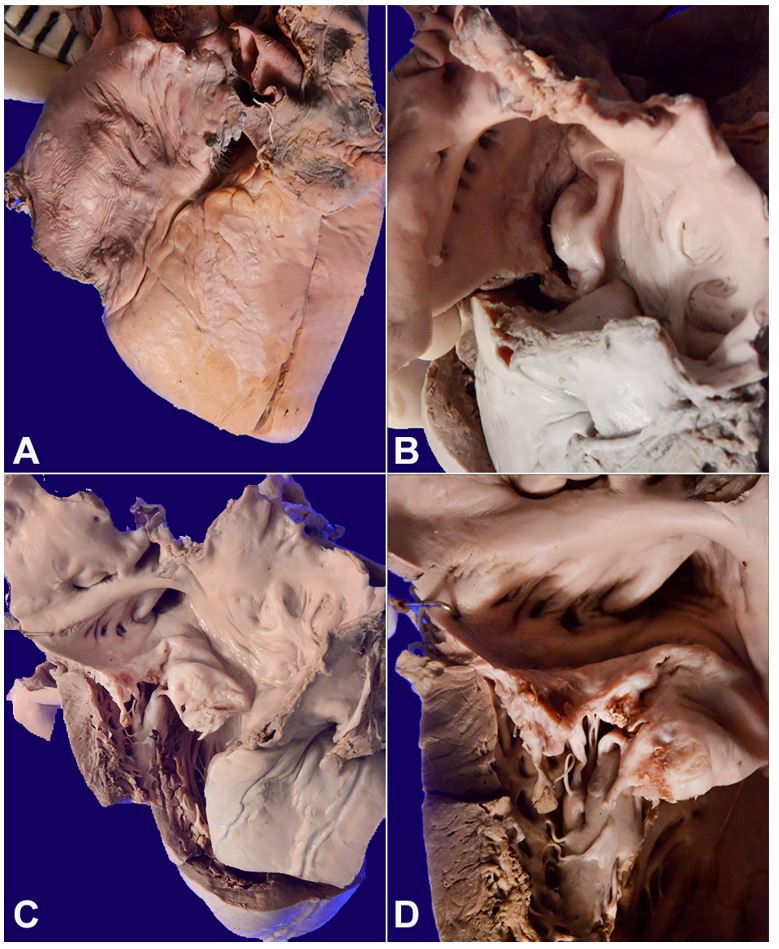
Gross view of the heart showing in **A** – Milk spots and soldier patches over the pericardium; **B** – severely stenosed mitral orifice simulating fish mouth appearance; **C** – dilated left atrial chamber with fibrotic and calcified mitral valve leaflets; **D** – friable vegetations in posterior mitral leaflet along lines of closure.

With due consent from the deceased relatives, a partial autopsy without examination of the brain was performed. Evisceration was done *in toto* with midline thoracoabdominal incision. On the opening of the serous cavities, the peritoneal cavity yielded 500 ml of straw-colored fluid. The heart weighed 320 g (RR; 233-383 g). The pericardial surface over the right atrium and right ventricle showed greyish white lesions, so-called Milk spots, and soldier’s patch, respectively (Figure 1A) and indicative of the healed phase of pericardial involvement in acute rheumatic fever. Both anterolateral and posterior-medial commissures were fused, and the mitral orifice was narrowed significantly and simulate a fish mouth (Figure 1B) when viewed through the left atrium. The heart was opened by the inflow/outflow method. Both anterior and posterior mitral leaflets were markedly thickened, calcified with erosion on their flow surface (Figure 1C). Two friable greyish white vegetations measuring 5 and 3 mm was noted in the posterior mitral leaflet along the lines of closure (Figure 1D). The aortic, tricuspid, and pulmonary valves were normal. The right ventricle wall was thickened and measured 8mm [RR;3-5 mm], indicating right ventricular hypertrophy, whereas the left ventricle wall thickness was in the normal range. On microscopy, two leaflets of the mitral valve shown calcification, both within the cusps and also over their surface, causing ulceration.

**Figure 2:**
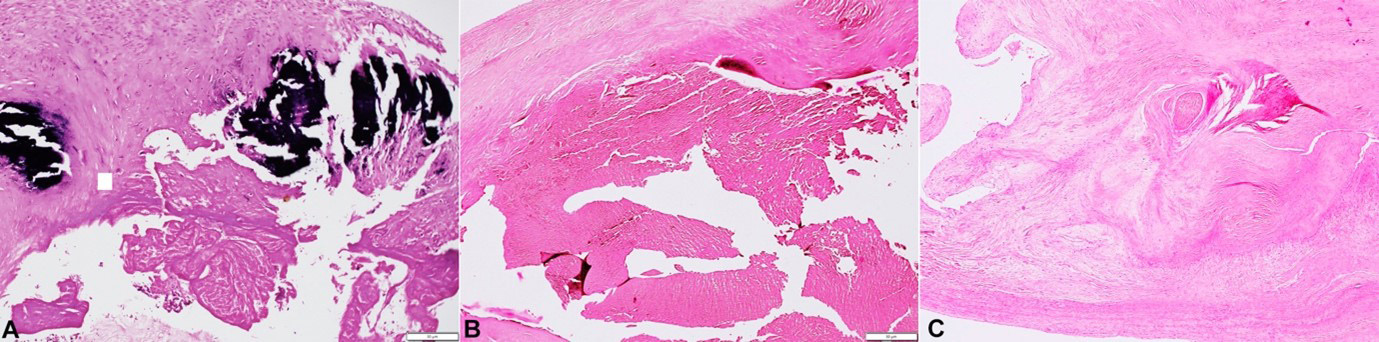
Photomicrographs of the mitral valve. **A** – Fibrotic valve with calcification both within posterior mitral valve leaflet and also over the surface causing ulceration and fibrin deposition. (H&E, 200X); **B** – Bland fibrin with devoid of any inflammatory cells densely adherent to leaflet margin. (H&E, 200X); **C** – Anterior Mitral valve leaflets showing features of classical chronic rheumatic valvulitis such as distorted layered architecture, myxoid degeneration and neovascularization. (H&E, 200X).

The histopathological examination of the suspicious vegetations showed bland fibrin adherent to the leaflets’ surface. There were no inflammatory cells noted in this fibrinous material (Figure 22B). Other areas of the mitral valve leaflets showed features of chronic rheumatic valvulitis such as dense fibrosis with myxoid degeneration, neovascularization (Figure 2C). There was no fungal profile seen and no organisms were demonstrated with special stains such as Gram, Gram Twort and PAS. ZN stain for AFB was negative. Considering both gross and microscopic findings, diagnosis of non-infective thrombotic endocarditis was made.

**Figure 3:**
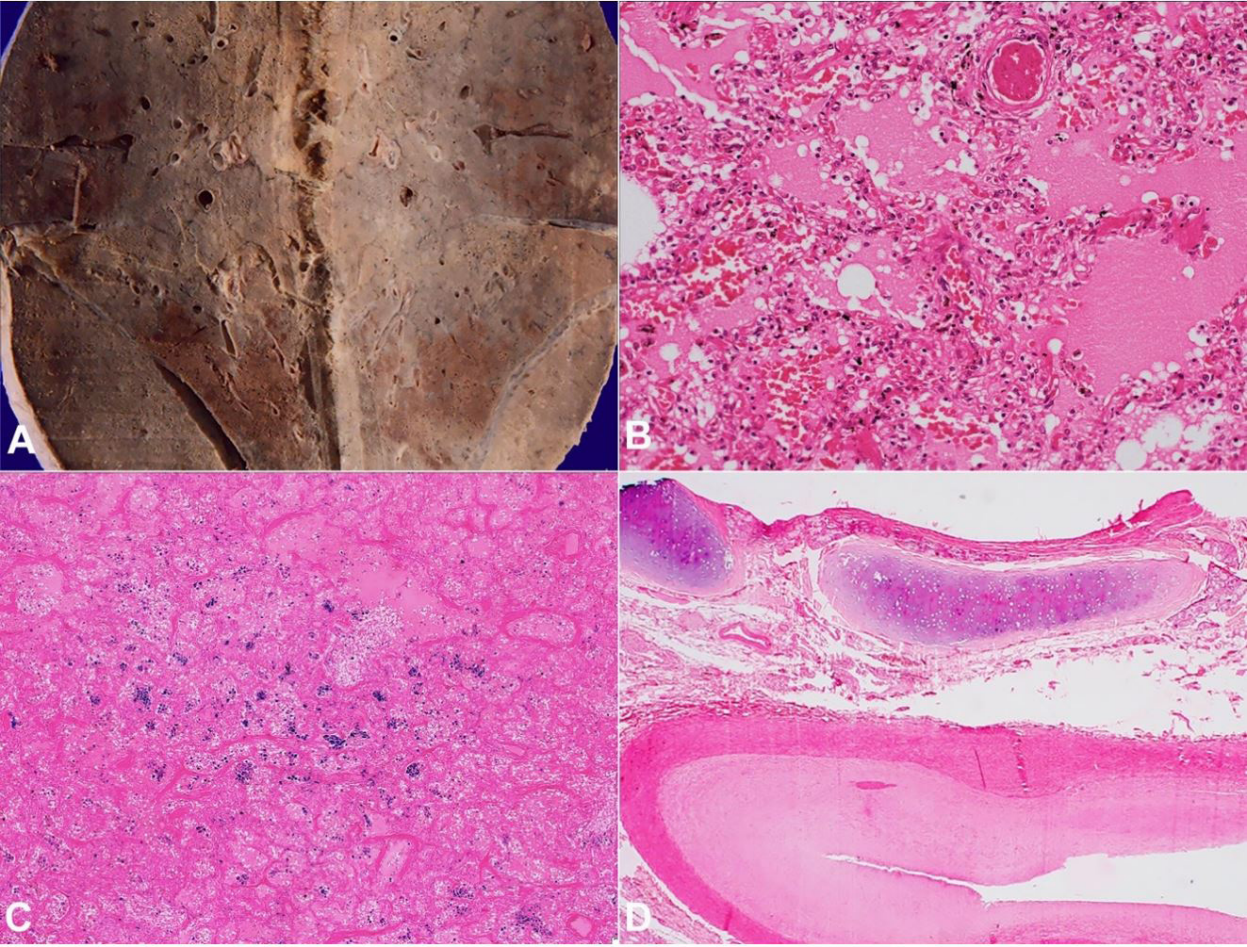
**A** – Gross view of the lung showing brown induration predominantly involving the lower lobe and few greyish white nodular lesions just beneath the thickened pleura; **B** – photomicrograph of the lung showing congested capillaries within the interalveolar septa with extravasation of erythrocytes and edema in the alveolar spaces. (H&E 200X), **C** – Perl's stain highlighting the presence of hemosiderin laden macrophages within alveoli (100X), **D** – Pulmonary artery showing eccentric intimal hyperplasia and medial hypertrophy (H&E, 200X).

**Figure 4:**
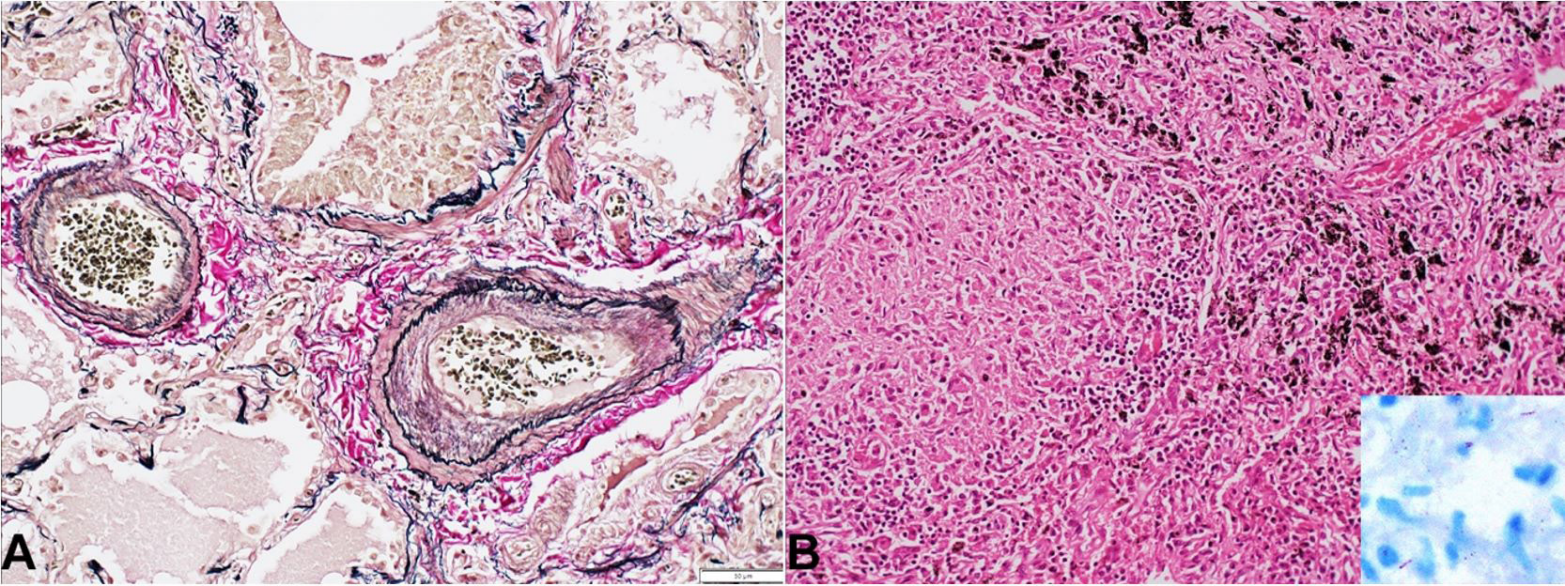
Photomicrograph of the lung. **A** – prominent inter and pre-acinar pulmonary arteries with concentric intimal hyperplasia and medial hypertrophy. (Elastic Van Gieson, x 200); **B** – Histological examination of greyish white lesions showing hyalinized intra-alveolar granulomas with giant cells. (H&E, 200X). Inset: Ziehl Nielsen stain showing the presence of acid-fast bacilli. (Oil immersion).

Both lungs weighed 1650 g (RR; 1100-1500 g) and the pleura was densely adherent to the chest wall and diaphragm. The cut lung’s surface was consolidated and showed brown induration. Besides, few greyish white lesions measuring 4 to 8 mm were noted in both lungs (Figure 3A). The microscopic examination of the lungs showed extensive vascular and interstitial remodeling changes of chronic passive venous congestion due to severe mitral stenosis such as increased and congested capillaries with extravasated RBCs (Figure 3B) and pigmented laden macrophages in alveolar spaces (Figure 3C); arterialization of veins; marked eccentric intimal hyperplasia and medial hypertrophy of pulmonary artery (Figure 3D); prominent inter and pre-acinar pulmonary arteries with concentric intimal hyperplasia (Figure 4A). Histological examination of the greyish-white lesions showed hyalinized intra-alveolar granulomas with giant cells (Figure 4B). Hilar lymph nodes also showed multiple granulomas with areas of necrosis. ZN stain for AFB was positive (Figure 2F inset).

**Figure 5:**
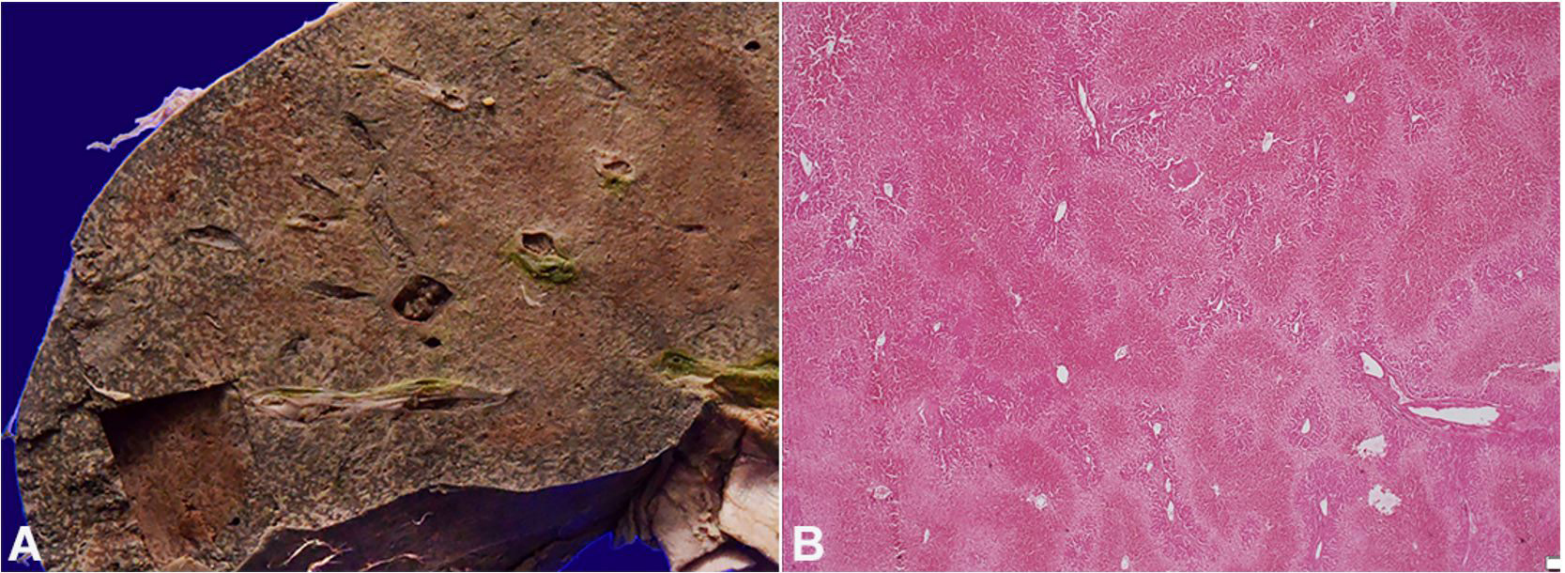
**A** – Gross view of the cut surface of the liver showing alternate dark and pale areas (Nutmeg liver), **B** – photomicrograph of the liver showing centri-zonal hemorrhagic necrosis with preserved periportal hepatocytes. There is no Porto-portal or Porto-central bridging fibrosis. (H&E 100 X).

**Figure 6:**
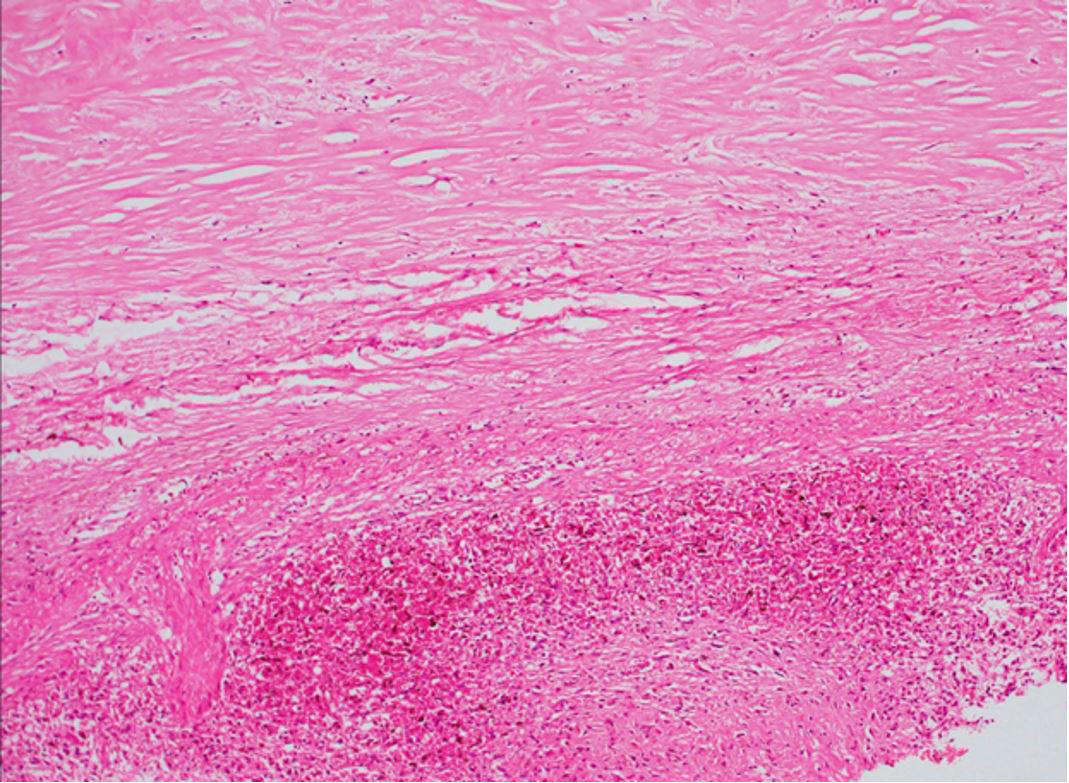
Microscopy of spleen showing thickened capsule and hyalinized granulomas. (H&E, 200X).

The liver weighed 995 g (RR; 968-1860 g) and showed classic features of congestive hepatopathy. The liver’s cut surface showed alternate dark and pale areas (Nut-meg appearance) (Figure 5A), which on microscopy shown centri-zonal hemorrhagic necrosis with preserved periportal hepatocytes (Figure 5B). No porto-portal or porto-central bridging fibrosis was seen. The spleen was congested with the thickened capsule, which on microscopy revealed hyalinized granulomas (Figure 6). The prostate and epididymis also showed necrotizing granulomatous inflammation. The kidney, bladder, stomach, intestine, pancreas, adrenals, thyroid were normal.

## DISCUSSION

Non-infectious thrombosis of the endocardium is one form of vegetation on cardiac valves histologically characterized by fibrin and platelet aggregates devoid of inflammation and bacteria.^2^ It is increasingly recognized in the autopsy. The reported incidence varies from 0.3 to 9.3%.^3^ In a large autoptic series by Bussani et al,^4^ the incidence of NBTE in the autopsy was found to be 3.7% and higher than that of infective endocarditis (1.1%). They also observed that when compared to infective endocarditis, NBTE was more frequently developed on pre-existing valvular lesions, including rheumatic heart disease (35% vs. 13%). Malignant neoplasm, particularly mucinous adenocarcinoma, pneumonia, cirrhosis, autoimmune disorders, and hypercoagulable state, are the most common associations found in that study.

The index case had shown classic complications and sequela of chronic rheumatic mitral valvular heart disease. such as features of pulmonary hypertension due to congestive vasculopathy, congestive hepatopathy. and splenomegaly in the autopsy. Though culture-negative infective endocarditis was kept during the patient’s evaluation, bland vegetation with no inflammatory cells was seen along lines of closures of the mitral leaflets. These autopsy findings are very characteristic of NBTE. In the absence of any other autoimmune disease or malignancy, NBTE seen in this case was possibly attributed to tuberculosis. Only a few studies in the literature document the association of tuberculosis with NBTE. In autopsy series study of NBTE by Llenas-García et al,^5^ tuberculosis was associated in 3 cases (13.6% of total), of which one case also had systemic lupus erythematosus. In the post-mortem imaging study by Noriki et at,^6^ one case with military tuberculosis also showed NBTE involving the aortic valve as a cause for emboli causing cerebral infarction. Though the precise pathogenetic association between tuberculosis and NBTE is not clear, endothelial damage and exposure of the subendothelial collagen to platelets as a result of septicemia may be the basic mechanism for the formation of thrombi over leaflets.

As the patient did not have classic symptoms of pulmonary tuberculosis except for fever, which was misinterpreted as due to the cardiac vegetations. Also, there was no clinically detected lymphadenopathy, and therefore, tuberculosis was not suspected during the hospitalization. Further, a chest X-ray done failed to demonstrate focal lesions in the lungs as observed during the autopsy. Probably, extensive vascular and parenchymal remodeling changes occurred in the lungs as a sequala of severe mitral stenosis masked the focal lesions in the Chest X-ray.

Clinically, infective endocarditis and nonbacterial thrombotic endocarditis may have overlapping signs and symptoms. A chance of misdiagnosis may be higher if the latter present with fever as occurred in this case. Though microbiological culture can be negative in infective endocarditis, in cases of repetitive negative cultures and patients not responding to broad-spectrum antibiotics, the possibility of NBTE should be considered, and another diagnosis has to be vigorously searched.

## CONCLUSION

Autopsy materials are a great source of learning. Symptoms of infections with vegetations in the valve on echocardiogram may not always represent infective endocarditis. It may also be seen in NBTE due to associated other infections in developing countries. Considering the increased incidence of NBTE than infective endocarditis in autopsy material, the differential diagnosis of NBTE should also be kept in mind strongly in evaluating vegetations in cases of rheumatic heart disease.
